# Effectiveness of a pharmacist-delivered smoking cessation program in the State of Qatar: a randomized controlled trial

**DOI:** 10.1186/s12889-017-4103-4

**Published:** 2017-02-20

**Authors:** Maguy Saffouh El Hajj, Nadir Kheir, Ahmad Mohd Al Mulla, Rula Shami, Nadia Fanous, Ziyad R. Mahfoud

**Affiliations:** 1 Clinical Pharmacy and Practice Section, College of Pharmacy, Qatar University, P.O. Box 2713, Doha, Qatar; 2 Tobacco Control Unit, Medicine Department, Hamad Medical Corporation, Doha, Qatar; 3 Department of Healthcare Policy and Research, Weill Cornell Medicine- Qatar, P.O. Box 24144, Doha, Qatar

**Keywords:** Smoking cessation, Pharmacist, Qatar, Intervention, Tobacco cessation, Randomized controlled trial

## Abstract

**Background:**

Cigarette smoking is one of the major preventable causes of death and diseases in Qatar. The study objective was to test the effect of a structured smoking cessation program delivered by trained pharmacists on smoking cessation rates in Qatar.

**Methods:**

A prospective randomized controlled trial was conducted in eight ambulatory pharmacies in Qatar. Eligible participants were smokers 18 years and older who smoked one or more cigarettes daily for 7 days, were motivated to quit, able to communicate in Arabic or English, and attend the program sessions. Intervention group participants met with the pharmacists four times at 2 to 4 week intervals. Participants in the control group received unstructured brief smoking cessation counseling. The primary study outcome was self-reported continuous abstinence at 12 months. Analysis was made utilizing data from only those who responded and also using intent-to-treat principle. A multinomial logistic regression model was fitted to assess the predictors of smoking at 12 months. Analysis was conducted using IBM-SPSS® version 23 and STATA® version 12.

**Results:**

A total of 314 smokers were randomized into two groups: intervention (*n* = 167) and control (*n* = 147). Smoking cessation rates were higher in the intervention group at 12 months; however this difference was not statistically significant (23.9% vs. 16.9% *p* = 0.257). Similar results were observed but with smaller differences in the intent to treat analysis (12.6% vs. 9.5%, *p* = 0.391). Nevertheless, the daily number of cigarettes smoked for those who relapsed was significantly lower (by 4.7 and 5.6 cigarettes at 3 and 6 months respectively) in the intervention group as compared to the control group (*p* = 0.041 and *p* = 0.018 respectively). At 12 months, the difference was 3.2 cigarettes in favor of the intervention group but was not statistically significant (*p* = 0.246). Years of smoking and daily number of cigarettes were the only predictors of smoking as opposed to quitting at 12 months (*p* = 0.005; *p* = 0.027 respectively).

**Conclusions:**

There was no statistically significant difference in the smoking cessation rate at 12 months between the groups. However, the smoking cessation program led to higher (albeit non-significant) smoking cessation rates compared with usual care. More research should be conducted to identify factors that might improve abstinence.

**Trial registration:**

Clinical Trials NCT02123329. Registration date 20 April 2014

## Background

Tobacco use is one of the major public health threats in the world [1]. It is responsible for killing globally around six million people a year, and current trends indicate that this number will increase to more than eight million deaths per year by 2030 [2]. Cessation of tobacco use is associated with significant health benefits [3]. The World Bank suggests that around 180 million tobacco related deaths could be prevented between now and 2050 if adult tobacco consumption decreased by 50% by 2020 [4]. However, quitting tobacco use can be very challenging without assistance from healthcare professionals. Evidence confirms that tobacco cessation interventions provided by healthcare practitioners are more effective when compared to self-help [5]. Pharmacists are easily accessible by the public and pharmacologic tobacco cessation aids have become increasingly available over the counter in pharmacies. Accordingly, pharmacists have a great opportunity to play a significant tobacco cessation role in the community [6, 7]. Pharmacist-provided tobacco cessation services in the community and ambulatory settings have been described in the literature, and these included randomized controlled trials, before-and-after studies, or single arm studies. Tobacco cessation services which included individualized counseling or within-group sessions have resulted in better smoking abstinence as compared with usual care. Abstinence has been either self-reported or biochemically verified using exhaled carbon monoxide, salivary or urinary cotinine levels [7–14].

The International Pharmaceutical Federation (FIP) published a statement of policy that outlines the anticipated role of pharmacists in eliminating the use of tobacco in the communities they serve. As per this statement, the pharmacist should provide tobacco cessation services to anyone who is considering quitting tobacco use or to anyone who suffers from tobacco-induced diseases [15]. The EuroPharm Forum in collaboration with the World Health Organization (WHO) Tobacco Unit issued a practical guide in which pharmacists were encouraged to become involved in tobacco cessation through helping and motivating people to quitting smoking, to offering extensive smoking cessation services [16]. The clinical practice guidelines for treating tobacco use and dependence developed by the U.S. Department of Health and Human Service recommended that clinicians (including pharmacists) intervene with any patient who uses tobacco [5]. In the American Society of Health-System Pharmacists’ (ASHP) Therapeutic Position Statement on the Cessation of Tobacco Use, healthcare providers, including pharmacists, were highly endorsed to improve the health of their patients by incorporating into their daily practice the identification of patients who use tobacco and the delivery of tobacco cessation services [17].

Tobacco use is one of the major preventable causes of death and disease in Qatar. According to the 2013 Global Adult Tobacco Survey (GATS), 20.2% of men in Qatar currently smoke tobacco and more than two fifths (41.5%) of current smokers report having their first smoke within half an hour of waking up [18]. In addition, 22.8% of boys aged 13 to 15 years currently use some form of tobacco products as per the 2013 Global Youth Tobacco Survey (GYTS) [19]. Notably, tobacco related diseases, including cardiovascular diseases (CVDs), are very common in Qatar. Ischemic heart disease is the leading cause of death in Qatar, accounting for 0.4 thousand deaths in 2012 [20]. Moreover, in the period from 1991 and 2006, lung cancer was ranked as one of the leading cancers in men with an incidence rate of 5.9 per 100,000 population [21]. It has been reported that on the average, 65 million dollars are spent on cigarettes per year in Qatar with 150 million dollars spent annually to cover the healthcare cost of smoking related diseases [22]. To decrease tobacco use, Qatar government has endorsed the Framework Convention on Tobacco Control (WHO FCTC) and has implemented several tobacco control activities [23]. There is a great opportunity for Qatar pharmacists to combat this public health burden. More than 700 pharmacists are currently working in ambulatory care clinics and in community pharmacies in Qatar. Nevertheless, ambulatory and community pharmacists in Qatar are not entirely involved in tobacco cessation activities. Only 21% of pharmacists in Qatar frequently ask patients about their smoking status, only 47% offer counseling to nicotine replacement therapy (NRT) purchasers, but more than 80% are interested in providing smoking cessation counseling [24]. Qatar pharmacists’ positive attitudes toward smoking cessation counseling can be translated into action if there is any existing smoking cessation model that pharmacists can utilize in their day to day practice. However, to date an intensive patient-specific program of smoking cessation that is designed exclusively for implementation in community and ambulatory pharmacies in Qatar does not exist.

The current study’s objective was to test the effect of a face-to-face structured patient-specific smoking cessation program delivered by trained ambulatory pharmacists on smoking cessation rates in Qatar.

## Methods

The study methodology is available in details in the published study protocol [25].

### Study design

The study was a prospective randomized controlled trial that compared the effectiveness of a face-to-face structured patient-specific smoking cessation program conducted by trained ambulatory pharmacists with brief unstructured pharmacist-delivered advice on smoking cessation rates.

### Study setting

The study was implemented in eight public and private ambulatory pharmacies in Qatar. Two pharmacists from each site were invited to participate in the study.

### Study pharmacists’ training

Before starting the study, a copy of the study methodology was sent to each study pharmacist along with literature on smoking cessation. Three weeks later, the study pharmacists attended a 2-day (8 h/day) smoking cessation training workshop.

The workshop was organized by the Hamad Medical Corporation (HMC) smoking cessation clinic team and Qatar University (QU) College of Pharmacy. The training team consisted of a public health and disease control consultant (who is also the HMC smoking cessation clinic head), academic faculty members with expertise and interests in tobacco cessation from teaching and research perspectives, and an international expert in the area of tobacco cessation.

The workshop included the following elements: smoking epidemiology and risks, benefits of quitting, transtheoretical model for behavior change, behavioral modification techniques, classification of smokers according to their stage of change, NRT, patient counseling techniques, development of a personalized action plan, program methodology and other needed elements.

In addition, the study team went over the study protocol with the pharmacists including participant group allocation, how to deliver the intervention and what to do with control patients.

Pharmacists were trained to help smokers identify the situations that may trigger craving to smoke and to provide them with strategies to improve coping [26]. Moreover, the pharmacists were educated to provide the smokers with information on what to expect during quit attempts and to address any concerns that patients may have [27].

Pharmacists were further trained on how to document the patient’s smoking status, medical and medication history, basic demographic characteristics, stage of change, education, follow-up and the agreed personalized action plan to quit smoking. In addition, they were taught how to fill the Fagerstrom test for nicotine dependence (FTND). The FTND is a six-item questionnaire designed to determine a patient’s degree of nicotine dependence [28]. This questionnaire has already been used in smoking-related studies in the Middle East [29, 30].

### Study advertising

The study pharmacists were encouraged to identify any opportunity to promote the smoking cessation program. For instance, they were advised to ask patients about their smoking status and to inform them about the smoking cessation program when filling prescriptions. Furthermore, study pharmacists were provided with posters and leaflets to display at their sites or to hand to clients. Moreover, letters were sent to the general practitioners and dentists practicing in the study’s ambulatory clinics inviting them to refer to the pharmacy any smoker who expresses interest (or is considering) to quit smoking.

### Screening and eligibility criteria

Smokers who were interested in quitting met with the study pharmacists who in turn assessed their eligibility for inclusion in the study. The screening and enrollment of participants was done by the study pharmacists at the sites on a patient by patient basis. Eligible participants were any patients aged 18 years and older who smoked one or more cigarettes daily for 7 days, were motivated to quit, were able to communicate in Arabic or English, and were willing and able to attend the scheduled sessions at the study pharmacies. The motivation to quit was determined by the pharmacist using the transtheoretical model of behavior change [31].

Exclusion criteria were (1) use of other nicotine or tobacco products; (2) current use or use in the last 30 days of smoking cessation aids or medications; (3) planning to leave Qatar in the next 12 months; (4) presence of any major medical condition that would prevent use of the NRT; (5) pregnancy; and (6) psychiatric illness or another debilitating condition that would interfere with participation in the study.

### Study enrollment

After the screening process was completed, the study pharmacist provided the eligible participant with relevant information on the smoking cessation program and explained the potential benefits and risks of participation. Before enrollment in the study, the participant was requested to sign an informed consent form. Once this form was signed, the pharmacist scheduled an appointment with him or her at dates and times that were appropriate for both of them.

### Randomization

Randomization was made using a sealed envelope technique. Permuted block randomization method with block of size 2 and 4 was used. The sequences were generated by the study statistician using a computer program from the website randomization.com (http://www.randomization.com). Independent sequences were generated for the different study pharmacists. Serially numbered, opaque, sealed randomization envelopes were then provided to each study pharmacist. The study statistician was not involved in recruitment of participants or in data collection. The envelope with the lowest number of randomized treatment blocks was opened by the pharmacist upon inclusion of each new participant.

### Initial appointment

At the initial appointment, which took 30 min, the study pharmacist collected information regarding the participant’s sociodemographic characteristics, current medical problems and medications, smoking history and vital signs. In addition, the pharmacist measured the participant’s exhaled carbon monoxide (CO) level using CO smokerlyzer. Moreover, the pharmacist assessed his or her nicotine dependence using the FTND. The participant was then randomly assigned to one of two groups: intervention group or control group based on the randomization sequence that was anonymously set earlier by the study statistician for each study pharmacist. Participant was informed of his or her group allocation. A new appointment was scheduled with participants in the intervention group. Brief unstructured smoking cessation advice was given to participants in the control group.

### Intervention group

Smokers assigned to the intervention group participated in a face-to-face one to one four-session program at the pharmacy by the study pharmacist at 2 to 4-week intervals over 8 weeks.

#### First session

The first session took around 30 min. In this session, the study pharmacist facilitated the participant’s preparation to quit. The participant selected a quit date in the next 2–4 weeks. The pharmacist explained to the participant the benefits of smoking cessation and offered him/her tailored behavioral and lifestyle strategies. The pharmacist also discussed with the participant what to expect during the quit attempt and provided him or her with strategies to cope with the early days of quitting.

NRT was provided to the participant as a patch or lozenge based on his or her prior experience, preference and the medication side effect profile. Before the NRT was dispensed to the participant, the study pharmacist checked if the participant had any contraindications to nicotine therapy and was not currently using any medication that would interact with NRT. The participant started the NRT on his or her chosen quit date. The study pharmacist counseled the participant about the dosage regimen, administration, duration of therapy, adverse effects, drug interactions, and disposal of NRT.

Participants who were given the nicotine patch started at 21 mg if they smoked 10 cigarettes or more per day. Otherwise, they received a 14-mg patch. Both dosages were continued for 6 weeks before being tapered. The 21-mg patch was decreased to 14 mg, continued for 2 weeks and then decreased to 7 mg for the final 2 weeks. Patients who started with the 14-mg patch were tapered to 7 mg for the final 2 weeks [32]. Participants who preferred the nicotine lozenge used the 1-mg pieces for 6 weeks as one lozenge every 1–2 h before tapering. Then the participants took one lozenge every 2–4 h for weeks 7 to 9, then one lozenge every 4–8 h for week 10 to 12 [33]. The pharmacist also designed a personalized action plan for each participant.

#### Follow-up sessions

The first follow-up session was scheduled 1 week after the participant’s set quit day and took around 20 min. In this session, the pharmacist evaluated the participant’s smoking status and NRT tolerability and measured his or her vital signs and exhaled CO level. If the participant was successful in quitting, the pharmacist offered reinforcement and addressed any concerns related to quitting. If the participant failed to quit, the pharmacist cautiously assessed the participant’s quit attempt and targeted all recognized obstacles. If the participant did not tolerate the NRT, the pharmacist recommended that the participant would stop the NRT and would visit the HMC smoking cessation clinic for additional support. In this case, the participant was considered withdrawn from the study.

The second follow-up session was scheduled 2 weeks after the first follow-up session. The third follow-up session was set 4 weeks after the second follow-up session. In these sessions, which were 10 to 15 min long, the pharmacist assessed the participant’s smoking status, vital signs, exhaled CO level, NRT tolerability and provided NRT refills. In addition, the pharmacist used cognitive-behavioral strategies to prevent relapse. After the third and last follow-up session, the participant was invited to contact the pharmacist in case he or she needed any further support.

### Control group

Participants in the control group received 5 to 10 min of unstructured one to one brief smoking cessation counseling by the pharmacist emulating current practice. They were also provided with educational materials about smoking cessation and were offered NRT in case of no documented contraindications or drug-drug interactions. The NRT dosage regimen and duration of therapy were similar to the ones used in the intervention group. Control participants did not attend any follow-up sessions.

### Outcome measures

Research assistants, who were blinded to the study participants’ group allocation, contacted the participants by phone and assessed the following outcome measures at 3, 6 and 12 months post the participants’ enrollment dates in the study:

- Self-reported 7-day point prevalence abstinence, defined as having smoked no cigarettes for the previous 7 days
- Self-reported 30-day point prevalence abstinence defined as having smoked no cigarettes in the last 30 days
- Self-reported continuous abstinence defined as having smoked no cigarettes since quit day

To objectively determine long-term abstinence, at 12 months, participants who self-reported not smoking were invited to come to their study clinic to measure their exhaled CO level by the clinic nurse who was blinded to the participants’ group. Participants were considered abstinent if they register less than 6 ppm (parts per million) on the test [34].

In case the participants were still smoking at each assessment point, they were asked about the number of cigarettes smoked per day and about the reasons for not stopping using open ended questions.

The study primary outcome was self-reported continuous abstinence at 12 months. Secondary outcomes included: 7-day point prevalence, 30-day point prevalence and continuous abstinence at 3 months and at 6 months and 7-day point prevalence and 30-day point prevalence abstinence at 12 months.

### Data analysis

The CONSORT guidelines were followed when analyzing the study data [35]. Demographic characteristics and other smoking related variables were summarized using frequency distributions (for categorical variables) and means with standard deviations (for numeric variables) and were compared between the two groups using the chi-squared test (or alternatively the Fisher’s exact test for small cell counts) for categorical variables and the independent *t*-test (or alternatively the Wilcoxon rank sum test for non-normally distributed variables) for numerical variables.

The primary study outcome was self-reported continuous abstinence at 12 months. Verification by the CO test at 12 months was not used as part of the primary outcome as was planned in the original study protocol since only 8 participants returned for their CO measurements. The percentages of participants achieving the primary outcome were computed and compared between the two groups using the chi-squared test. Moreover, the difference in the proportions of continuous abstinence at 12 months between the two study groups (with 95% confidence intervals) was computed along with the number needed to treat (computed as 1 divided by the difference in the proportions of the primary outcome between the two study arms). Multiple logistic regression was used to obtain the odds ratio of the primary outcome between the two study groups adjusting for any imbalances in demographic or other variables. In addition, mixed effects logistic regression, with pharmacy-level random effect, was considered to adjust for the possible pharmacy effect if it existed. The main regression for the primary outcome was also adjusted for the number of monitoring visits that the participants attended to assess the adherence effect. The analyses of the primary and secondary outcomes were done using responders only and also using the intent-to-treat principle whereby participants who were lost to follow-up were classified as smokers.

Differences in abstinence between the study groups were also assessed at 3 and 6 months using the Chi-squared test or Fisher’s exact test. For those who relapsed at 3, 6 and 12 months, the number of cigarettes smoked daily was summarized using means and standard deviations and compared between the study groups in a similar way to other numeric variables described above.

According to the self-reported abstinence outcome at 12 months; participants were divided into 3 groups: abstainers, smokers and dropouts. Dropouts included those who were lost to follow up at 12 months. A multinomial logistic regression model was fitted with this new variable as the dependent variable and several independent variables including demographic, baseline smoking related variables and study group (similar to what is suggested by Borelli et al., 2002) [36]. The model allowed for the simultaneous detection of predictors for those who smoked and those who dropped out from the study. Results were summarized using Relative Risks Ratios along with 95% confidence intervals and *p*-values. Statistical analyses were conducted using the IBM-SPSS version 23 and STATA version 12. In all analyses, the level of significance was set at 5%.

### Sample size calculation

For a priori sample size calculation, we assumed a 7-day point prevalence abstinence at 12 months of 3% for the control group and 15% for the intervention group based on the results of one of the previous pharmacist-run smoking cessation studies. With a two-sided alpha of 5% and 90% power, a minimum sample size of 118 participants was estimated for each group.

### Continuous quality improvement and evaluation plan

At all stages of the study implementation, the study pharmacists were closely supervised by the study team. Supervision was through several mechanisms including meetings, communication and site visits.

## Results

**Fig. 1:**
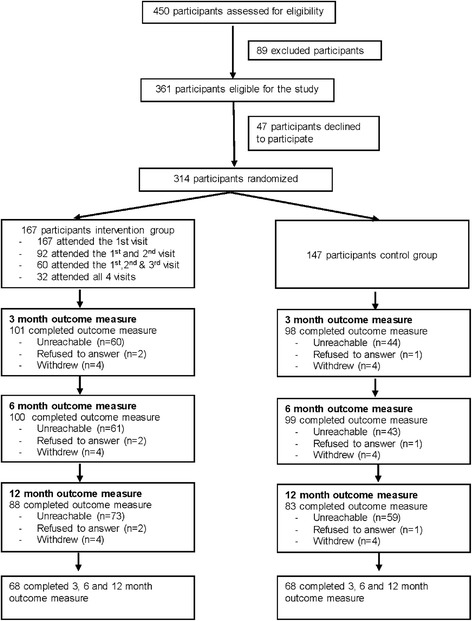
Participant flow chart

Between February 2013 and December 2014, the study pharmacists assessed 450 smokers for eligibility in the study. Of them, 361 (80.2%) met the study inclusion criteria. A total of 314 cigarette smokers consented to enroll in the study and were randomized into one of two study groups (Fig. 1). Of 167 participants in the intervention group, 55% completed two pharmacy visits. Two patients in the intervention group could not tolerate NRT and as a result they were excluded by the study pharmacists.

Out of the 314 recruited participants, 171 (54.5%) were assessed at 12 months follow up and 136 (43%) participants were assessed at 3, 6 and 12 months. Only 8 (6 in the intervention arm and 2 in the control arm) of the 35 participants who self-reported abstinence at 12 months had a final CO measurement taken (22.9%) and results showed that all those 8 participants were nonsmokers.

Participants were mainly men (97.8%) below the age of 40 years (59.3%), had been smoking for more than 10 years (68.4%), had a FTND score of more than 5 (61.1%) and had tried quitting before (69.5%). Except for nationality, previous quit attempts and having diabetes; demographic, tobacco related, and clinical variables were comparable between the two study groups (Table 1).

**Table 1:** Demographic and tobacco use variables compared between the two study groups

Variable	Intervention (*N* = 167)	Control (*N* = 147)	*p*-value
Age in years			0.221
18–29	40 (24.5%)	33 (23.2%)	
30–39	53 (32.5%)	55 (38.7%)	
40–49	46 (28.2%)	27 (19.0%)	
50 and above	24 (14.7%)	27 (19.0%)	
Gender (males)	164/167 (98.2%)	143/147 (97.3%)	0.710
Nationality			0.006*
Qatar	25 (15.4%)	36 (24.8%)	
Egypt	41 (25.3%)	49 (33.8%)	
Other	96 (59.3%)	60 (41.4%)	
Highest educational level			0.604
Primary	15 (9.3%)	16 (11.3%)	
Secondary/high school	40 (24.8%)	43 (30.3%)	
College diploma	40 (24.8%)	37 (26.1%)	
Undergraduate degree	49 (30.4%)	33 (23.2%)	
Post graduate degree	17 (10.6%)	13 (9.2%)	
Marital Status			0.389
Never married	32 (19.6%)	23 (15.9%)	
Ever married	131 (80.4%)	122 (84.1%)	
Years of cigarettes smoking			0.527
0 to 2.99 years	4 (2.4%)	7 (4.8%)	
3 to 4.99 years	13 (7.9%)	15 (10.3%)	
5 to 10 years	30 (18.3%)	29 (19.9%)	
More than 10 years	117 (71.3%)	95 (65.1%)	
Number of cigarettes per day			0.227
Mean(sd)	21.6 (11.9)	23.5 (13.6)	
Tried quitting before (Yes)	122/163 (74.8%)	92/145 (63.4%)	0.030*
Family member smokes (yes)	87/161 (54.0%)	80/145 (55.2%)	0.842
Chronic medical conditions			
Some chronic medical conditions	51/164 (31.1%)	31/145 (21.4%)	0.053
Hypertension	20/164 (12.2%)	15/145 (10.3%)	0.609
Diabetes	27/164 (16.5%)	12/145 (8.3%)	0.031*
Asthma/chronic lung diseases	6/164 (3.7%)	4/145 (2.8%)	0.755
Cardiovascular diseases	5/164 (3.0%)	3/144 (2.1%)	0.728
Gastrointestinal diseases	4/164 (2.4%)	4/145 (2.8%)	0.999
Depression	2/164 (1.2%)	0/145 (0.0%)	0.500
Physical Exercise			0.999
No	66 (41.8%)	59 (41.8%)	
Yes but irregularly	62 (39.2%)	55 (39.0%)	
Yes regularly	30 (19.0%)	27 (19.1%)	
Most important reasons for quitting†			
Religious reasons	55/129 (42.6%)	58/124 (46.8%)	0.508
To live healthier	88/152 (57.9%)	76/135 (56.3%)	0.785
To live longer	21/127 (16.5%)	25/121 (20.7%)	0.403
To be role model to children	30/132 (22.7%)	28/116 (24.1%)	0.793
Financial reasons	6/109 (5.5%)	6/100 (6.0%)	0.878
Pressure from health care providers	8/109 (7.3%)	11/106 (10.4%)	0.433
Baseline heart rate			0.810
Mean(sd)	79.0 (13.6)	79.3 (13.0)	
Baseline systolic blood pressure			0.626
Mean(sd)	126.0 (14.4)	125.1 (15.3)	
Baseline diastolic blood pressure			0.176
Mean(sd)	77.5 (10.5)	75.8 (10.5)	
Baseline weight			0.949
Mean(sd)	85.6 (17.7)	85.7 (20.2)	
Baseline Body Mass Index (BMI)			0.914
Mean(sd)	40.4 (32.9)	40.0 (27.9)	
Baseline FTND			0.828
Mean(sd)	5.3 (2.4)	5.0 (2.4)	

*significant difference between study arms † including those who gave all reasons same importance

The percentage of participants reporting abstinence at 12 months was slightly higher in the intervention group as compared to the control group, however this difference didn’t reach statistical significance (23.9% vs. 16.9%, Diff = 7.0%, 95% CI −5.2%, 18.9%, Number Needed to Treat = 15 and *p* = 0.257). When restricting the analysis to those who had complete data on all follow up assessments, a similar trend was observed but with a smaller difference between the study groups. Results of comparing continuous abstinence at 3 months and 6 months assessment showed lower differences between the two groups (Table 2). Intent to treat analysis for the primary outcome where participants with missing data were considered smokers also revealed nonsignificant difference between the two study arms (12.6% vs. 9.5% in the intervention arm and control arm respectively, *p* = 0.391, NNT = 33) (Table 2).

**Table 2:** Self-reported abstinence at each visit

	Responders			ITT		
Variable	Intervention	Control	*p*-value	Intervention	Control	*p*-value
Outcome measure at 3 months						
7 days abstinence	31/101 (30.7%)	26/98 (26.5%)	0.516	31/167 (18.6%)	26/147 (17.7%)	0.841
30 days abstinence	27/101 (26.7%)	22/98 (22.4%)	0.483	27/167 (16.2%)	22/147 (15.0%)	0.770
Continuous abstinence	25/101 (24.8%)	21/98 (21.4%)	0.578	25/167 (15.0%)	21/147 (14.3%)	0.864
Outcome measure at 6 months						
7 days abstinence	28/100 (28.0%)	21/99 (21.2%)	0.324	28/167 (16.8%)	21/147 (14.3%)	0.546
30 days abstinence	27/100 (27.0%)	20/99 (20.2%)	0.259	27/167 (16.2%)	20/147 (13.6%)	0.525
Continuous abstinence	23/100 (23.0%)	20/99 (20.2%)	0.632	23/167 (13.8%)	20/147 (13.6%)	0.966
Outcome measure at 12 months						
7 days abstinence	21/88 (23.9%)	14/83 (16.9%)	0.257	21/167 (12.6%)	14/147 (9.5%)	0.391
30 days abstinence	21/88 (23.9%)	14/83 (16.9%)	0.257	21/167 (12.6%)	14/147 (9.5%)	0.391
Continuous abstinence	21/88 (23.9%)	14/83 (16.9%)	0.257	21/167 (12.6%)	14/147 (9.5%)	0.391
Continuous abstinence at 12 months incorporating data from visits 3 and 6 and 12‡	9/68 (13.2%)	6/68 (8.8%)	0.412	9/167 (5.4%)	6/147 (4.1%)	0.588

‡this includes only participants who completed assessment of all outcome measures up to the time indicated

The main analysis did not change when adjusted for the possible clustering effect by the pharmacists, for observed imbalances in demographic and smoking related variables and for the number of attended monitoring visits, or when all those lost to follow up were considered smokers. For example, the unadjusted OR of 1.54 (95% CI 0.73, 3.29) changed to 1.62 (95% CI 0.65, 4.04) and *p* = 0.297 when it was adjusted for years of smoking, cigarettes per day, tried quitting before, diabetes and nationality (Table 3).

**Table 3:** Analysis of the primary outcome of continuous abstinence at 12 months

	Self-reported 12 months abstinence at the final visit		Continuous abstinence at 12 months (using all data from 3 visits)	
	OR (95% confidence interval)	*p*-value	OR (95% confidence interval)	*p*-value
Unadjusted OR	1.54 (0.73, 3.29)	0.259	1.58 (0.53, 4.70)	0.414
Adjusted OR^a^	1.54 (0.80, 2.99)	0.196	1.58 (0.45, 5.58)	0.480
Adjusted OR^b^	1.62 (0.65, 4.04)	0.297	1.86 (0.49, 7.07)	0.364
Adjusted OR^c^	1.60 (0.65, 3.87)	0.306	1.60 (0.43, 5.90)	0.483
Adjusted OR^d^	1.37 (0.67, 2.80)	0.393	1.34 (0.47, 3.85)	0.589

^a^adjusted for clustering effect within pharmacists ^b^adjusted for years of smoking, cigarettes per day, tried quitting before, diabetes and nationality ^c^adjusted for the number of monitoring visits the participants attended ^d^all those lost to follow up were considered as smokers

The average daily number of cigarettes smoked for those who relapsed was significantly lower by 4.7 and 5.6 cigarettes at 3 and 6 months respectively in the intervention group as compared to the control group. At 12 months, the intervention group smoked 3.2 cigarettes per day on average lower than the control group, but such difference was not statistically significant (Table 4).

**Table 4:** Changes in other tobacco related variables

Variable	Intervention Mean(sd)	Control Mean(sd)	*p*-value
Daily number of cigarettes at 3 months	16.1 (11.1) *N* = 67	20.7 (14.0) *N* = 62	0.041^a^
Daily number of cigarettes at 6 months	15.6 (11.9) *N* = 67	21.2 (14.1) *N* = 59	0.018^a^
Daily number of cigarettes at 12 months	16.9 (14.4) *N* = 61	20.1 (15.8) *N* = 59	0.246

^a^significant difference between the study groups

At 3 months, in the intervention group, stress at work or in life was the main stated reason for not stopping smoking (18.4%), followed by lack of motivation to quit (9.2%), inability to attend all the follow-up sessions (6.6%) and frequent travelling (6.6%). The most common stated reasons for not stopping smoking in the control group were stress at work or in life (26%), being around other smokers (6.5%) and frequent traveling (6.5%).

The only variables that were associated predictors of smoking as opposed to quitting at 12 months were years of smoking and number of cigarettes per day. In particular, the higher the number of years of smoking or the number of cigarettes smoked per day, the more likely the person relapsed and/or smoked at 12 months (RRR = 2.07, 95% CI 1.24, 3.45 p-value = 0.005 for Years of smoking). On the other hand, only years of smoking predicted dropping out as compared to quitting with similar trends as those seen for smoking compared to quitting (RRR = 1.89, 95% CI, 1.17, 3.06 *p*-value = 0.009) (Table 5).

**Table 5:** Predictors of smoking and dropouts at 12 months

Variable	Smoking RRR (95% CI)	*p*-value	Dropout RRR (95% CI)	*p*-value
Age (in years)				
18–29	1		1	
30–39	1.46 (0.38, 5.61)	0.584	1.58 (0.43, 5.83)	0.489
40–49	1.06 (0.25, 4.55)	0.934	0.66 (0.16, 2.79)	0.576
≥50	1.71 (0.29, 10.2)	0.554	1.85 (0.32, 10.56)	0.488
Marital Status				
Never Married	1		1	
Ever Married	0.50 (0.12, 2.08)	0.337	0.60 (0.15, 2.38)	0.465
Nationality				
Qatar	1		1	
Egypt	1.60 (0.49, 5.22)	0.432	0.58 (0.18, 1.84)	0.358
Other	1.60 (0.51, 5.03)	0.425	1.21 (0.41, 3.58)	0.734
Tried quitting before	0.77 (0.30, 1.99)	0.593	1.27 (0.49, 3.29)	0.619
Years of smoking	2.07 (1.25, 3.45)	0.005*	1.89 (1.17, 3.06)	0.009*
Cigarettes per day	1.04 (1.00, 1.09)	0.027*	1.01 (0.98, 1.05)	0.481
Study group	0.58 (0.24, 1.38)	0.218	0.70 (0.30, 1.64)	0.410

*significant association *p* < 0.05

## Discussion

This study was the first in Qatar, and probably in the Middle East, to assess the effectiveness of pharmacist-led smoking cessation program on smoking cessation rates. The study explored the potential role that Qatar’s primary health care pharmacists could play in promoting public health and disease prevention. Qatar pharmacists are always accessible to the public without the need of any prior appointment, and this allows them to oversee the patients’ progress in smoking cessation programs. The study helped to highlight the extent of contribution that they could have in addressing this public health burden in the country.

In the study, the smoking cessation rates in the intervention group were better than those in the control group; however this difference did not reach statistical significance. These findings are inconsistent with some other pharmacist led smoking cessation studies conducted elsewhere. For example, in one study from United Kingdom (UK), 484 smokers were randomly assigned to intervention and control groups in order to evaluate the effectiveness of a structured pharmacist-based smoking-cessation program compared with normal pharmaceutical service. At 12 months, 14.3% of smokers in the intervention group were abstinent compared with 2.7% in the control group (*p* < 0.001) [37]. In a study in Australia, smoking quit rates achieved by participants in a pharmacist-run smoking cessation program, which was started in a hospital and continued in either the community pharmacy or hospital setting, were compared with quit rates in a minimal intervention group. At 12 months, the 30-day point prevalence abstinence rate was 22.9% for the hospital intervention arm, 14.7% for the community pharmacy arm, and 3% for the control arm (*p* = 0.031) [8]. In another study conducted in the United States (US), smokers were randomly assigned to receive a 3-session face-to-face group program conducted by the pharmacist team or one 5- to 10-min standard care session delivered by the pharmacist team over the telephone. At 6 months, the biochemically confirmed smoking cessation quit rate was 28% in the face to face group and 11.8% in the standard care group (*p* < 0.041) [11].

Yet our results were similar to other studies. For instance, a study from Scotland evaluated the smoking quit rates of participants who received smoking-cessation counseling from trained pharmacists compared with standard pharmacy advice. At 9 months, the continuous self-reported smoking cessation rate was 11.6% in the intervention group and 7.1% in the control group (*p* = 0.089) [38]. Another study conducted in Australia assessed the effectiveness of a hospital pharmacist-led multi-component smoking cessation program versus usual hospital care. The difference in abstinence rates between intervention and control groups was not statistically significant at 6 (11.6 versus 12.6%) or 12 months (11.6 versus 11.2%) [39].

Many factors could have contributed to the lack of significant difference in smoking cessation rates between the intervention and control groups in the study. One such factor might be related to the pharmacy practice environment in Qatar, where the pace of advancement in community and ambulatory pharmacy practice has been slow. With very few pharmaceutical care activities, the scope of practice in Qatar is confined to filling prescriptions and dispensing medications; although this is slowly, but surely, changing [40]. Pharmacy is still perceived by the public as a profession that mainly focuses on medication supplies and accessibility. To change this perception and to recognize the pharmacist as a healthcare professional with a distinctive set of expertise and abilities, more work has to be done; and Qatar’s Health Strategy has already started steps towards launching programs leading in that direction.

In addition, although the intervention pharmacists received a two day training workshop on smoking cessation, given the different educational backgrounds of Qatar pharmacists and the cultural diversity of smokers in Qatar, it seems that providing a more intensive smoking cessation training with more rigorous follow-up and mentorship would be warranted. This recommendation is in line with one of the actions of Qatar Ministry of Public Health’s Tobacco Cessation Action Plan, which is capacity building and training of healthcare professionals on counseling and care for tobacco users [23]. The proposed training program should encompass aspects of behavioral and pharmacological therapies and should address elements of a personalized smoking cessation action plan that takes into consideration the smoker’s cultural background and his or her individual reasons for quitting.

Although the study pharmacists reminded participants several times about their follow-up visits, only 19% of intervention participants attended all four visits as originally planned in the study protocol. This may have decreased the effectiveness of the intervention as clinical practice guidelines report that the more intensive the treatment intervention is in duration or in number of attended sessions the better is the smoking cessation rate [5].

In addition, 61% of study participants had FTND score of more than 5 which indicates high nicotine dependence. Evidence suggests that high nicotine dependence is negatively associated with making a quit attempt and is one of the risk factors for relapse [41]. This may explain in part the relatively low smoking cessation rates seen in the study. The participants may have needed very specialized interventions, a more intensive treatment or other potent smoking cessation aids such as Varenicline and a closer follow-up by the pharmacist to stop smoking.

An additional factor that might have contributed to the insignificant differences in smoking cessation rates between the two groups could be the fact that the study pharmacists delivered care to participants in both intervention and control groups. It is plausible that the pharmacists, who were overall extremely motivated and enthusiastic, might have inadvertently contaminated the usual care group with extra care. This may have increased the smoking cessation rates in this group.

Another potential factor could be related to the study pre-calculated sample size and loss of follow-up rates. The sample size in the study was estimated based on the assumption of a 7-day point prevalence abstinence at 12 months of 3% for the control group. The abstinence rate at 12 months for the control group in the study was 16.9% which is much higher than what has been previously estimated. In addition, 47.3% and 43.5% of patients were lost to follow-up in intervention and control groups respectively at 12 months. This may have reduced the study power and resulted in the lack of statistically significant difference in smoking cessation rates between the two groups. The high rate of loss of follow-up in the study is not surprising. In spite of the tremendous efforts of the study research assistants to contact participants in both groups, many participants were not reachable on the phone. However, there were no differences in the demographic or smoking related variables between those who dropped and those who remained in the study except for nationality. In particular, the percentage of dropouts among Qataris and other nationalities was significantly higher than that among Egyptians (51.2% vs 31.1% respectively). This observed difference might have minimal to no impact on the study results since adjusting for nationality and other variables produced no considerable change in the significance level and the OR of the main comparison between the study arms. Moreover, after adjusting for other variables, nationality was not a predictor of dropping out of the study. Yet this information is important for researchers in future studies as they have to exert more efforts with Qatari participants and participants of other nationalities to decrease their dropout rates.

The top reason for continuing smoking in the intervention group was stress at home or work. This is consistent with findings from other pharmacist delivered smoking cessation programs [42, 43]. A more intensive and personalized approach by the pharmacist that includes strategies on coping with stress may have improved the smoking cessation rates in this group. The number of cigarettes per day and the years of smoking were found to be predictors of continuing smoking and relapse at 12 months. This finding is consistent with previous studies that confirmed how challenging smoking cessation is for smokers with high nicotine dependence [41–44]. This would mean that a more intensive intervention that could include collaboration with healthcare providers from different disciplines, intense behavioral therapy and more effective pharmacotherapy would have enhanced cessation rates in this group of smokers.

In spite of the finding that the pharmacist-led smoking cessation program was not significantly better in achieving smoking cessation rates compared with the control group, the self-reported smoking cessation rates in the intervention arm were 24.8%, 23% and 23.9% at 3, 6 and 12 months respectively. These rates are similar to those of other pharmacist led smoking cessation programs conducted elsewhere [11, 13, 45, 46].

Furthermore, the smoking cessation rates obtained in this study were higher than those obtained in smoking cessation programs headed or facilitated by other healthcare professionals including physicians [47, 48]. In addition, there was a significant reduction in the number of cigarettes smoked daily between intervention and control groups at 3 and 6 months. Therefore, despite failing to detect a significant difference between the intervention and the control groups, the study demonstrated that a pharmacist-led smoking cessation program can be implemented in Qatar and may hold promise with some adjustments. The generated data may provide the impetus to explore expanding the scope of pharmacy practice in the country and for involving pharmacists in providing smoking cessation counseling. As a follow-up to this study, a qualitative study using semi-structured interviews will be conducted with the study pharmacists and a sample of the study participants in the intervention arm to gain a deeper insight into the reasons behind the lack of significant difference between the two groups, the reasons for loss to follow-up, the barriers and challenges of the smoking cessation program and the elements of an effective pharmacist delivered smoking cessation program in Qatar.

### Limitations

The study had important limitations that must be considered. A substantial number of participants had missing data on follow-up visits. Of a total of 314 patients participated in the program; data were available for only 171 participants at 12 month follow-up visit. Only 8 of the 35 participants who self-reported abstinence at 12 months had a final CO measurement as a result CO measurement was not included in the main outcome assessment. There is a possibility of a ‘Hawthorne effect’ which may have overestimated the self-reported quit rates of participants in the usual care group.

Despite these limitations, the study had several strengths including the use of a randomized control design, long follow-up duration and use of intent-to- treat analysis.

## Conclusions

A structured smoking cessation program with intensive follow-up of smokers by trained ambulatory pharmacists in Qatar has proven to be more effective than usual care in reducing the number of daily smoked cigarettes at 3 and 6 months from the start of the program. However, this reduction was not statistically significant after 12 months of follow-up. The self-reported smoking cessation rates were higher in the structured program as compared with the usual care group; but this difference in rates between the groups didn’t reach statistical significance. The results clearly illustrate the difficulty faced by tobacco smokers to quit smoking for a prolonged time despite structured and intensive efforts by pharmacists. This should not undermine the role of pharmacist in smoking cessation in Qatar and what could be achieved in reducing the smoking rate through intensive follow-up and monitoring from pharmacist. More efforts should be exerted to intensify this program and to maximize the abstinence rate.

## Abbreviations

ASHP: American Society of Health-System Pharmacists
CO: Carbon Monoxide
FCTC: Framework Convention on Tobacco Control
FIP: International Pharmaceutical Federation
FTND: Fagerstrom test for nicotine dependence
HMC: Hamad Medical Corporation
IRB: Institutional Review Board
NRT: Nicotine Replacement Therapy
PPM: Parts per million
QU: Qatar University
UK: United Kingdom
US: United States
WHO: World Health Organization
